# Private Equity–Acquired Residential Treatment Facilities vs Other For-Profit Facilities

**DOI:** 10.1001/jamahealthforum.2026.0414

**Published:** 2026-04-03

**Authors:** John L. Havlik, Susan Busch, Kalea Hidalgo, Kimberly Mercado, David Zhu, Samuel Jensen, Jane M. Zhu

**Affiliations:** 1 Yale University School of Medicine, New Haven, Connecticut; 2 Yale School of Public Health, New Haven, Connecticut; 3 Division of Biological Sciences, University of Chicago, Illinois; 4 School of Medicine, Virginia Commonwealth University, Richmond; 5 School of Medicine, Oregon Health & Science University, Portland; 6 Division of General Internal Medicine and Geriatrics, Oregon Health & Science University, Portland

## Abstract

**Importance:**

Amid an ongoing addiction crisis, private equity (PE) firms have acquired large numbers of residential substance use disorder (SUD) treatment facilities in the US. How PE ownership affects SUD care prices and facility characteristics is not well understood.

**Objective:**

To evaluate prices and facility characteristics of residential substance use treatment facilities owned by PE firms compared with other for-profit facilities.

**Design, Setting, and Participants:**

This cross-sectional study using a secret shopper framework was conducted from June 2024 to April 2025. PE-acquired residential SUD treatment facilities were matched to non–PE-acquired, for-profit control facilities by geographic proximity. Trained callers posed as individuals seeking residential SUD care for a family member with pending Medicaid insurance.

**Exposures:**

Ownership by PE vs non-PE for-profit entities.

**Main Outcomes and Measures:**

The primary outcome was daily rate. Secondary outcomes included bed availability and wait times, admission requirements, services, staffing, amenities, and facility-initiated contact.

**Results:**

This study included 341 residential facilities with completed call data (127 PE-acquired, 214 geographically matched non-PE for-profit). Mean (SD) daily rates were 15.6% higher at PE facilities ($910.73 [$463.16]; median, $854.29 [IQR, $589.29-$1071.43]) compared with non-PE facilities ($779.87 [$501.92]; median, $750.56 [IQR, $480.64–$952.38]; Benjamini-Hochberg–adjusted *P* = .04). This significantly higher daily rate was also found after adjustment for geographic cluster-matched fixed effects (β = $127.73; 95% CI, $29.57–$225.87; adjusted *P* = .03). Compared with non–PE-acquired control facilities, PE-acquired facilities were less likely to offer detox services (74.8% vs 88.8%; adjusted *P* = .02) and private rooms (12.1% vs 25.7%; adjusted *P* = .02), and more likely to make postcall contact attempts (mean [SD], 0.68 [1.39] vs 0.18 [0.47]; adjusted *P* < .001).

**Conclusions and Relevance:**

In this national secret shopper study, PE-acquired residential SUD treatment facilities reported higher daily prices than geographically matched non-PE for-profit facilities. Price differences persisted after accounting for local market factors, suggesting systematic cost differences associated with ownership structure. This study provides facility-level evidence on pricing differences by ownership type in residential SUD treatment and highlights the need for further research to understand the mechanisms underlying these differences and their implications for patients and payers.

## Key Points

**Question:**

How do the price and facility characteristics of residential substance use treatment facilities owned by US private equity (PE) firms compare with other for-profit facilities?

**Findings:**

In this cross-sectional study including 341 residential facilities, mean daily rates were 15.6% higher at PE facilities compared with non-PE facilities. Compared with non–PE-acquired control facilities, PE-acquired facilities were less likely to offer detox services (74.8% vs 88.8%) and private rooms (12.1% vs 25.7%), and more likely to make postcall contact attempts.

**Meaning:**

Results of this study suggest that, as PE continues to expand its presence in behavioral health, understanding these dynamics and their impacts will be important for addressing an ongoing national substance use crisis.

## Teaser

This cross-sectional study compares prices and facility characteristics of residential substance use facilities owned by private equity firms with other for-profit facilities and whether there are differences by ownership type.

## Introduction

Private equity (PE) firms have increasingly targeted behavioral health facilities, including those treating substance use disorders (SUDs), amidst a national crisis of addiction and unmet treatment need.^1,2^ The behavioral health sector and, in particular, SUD treatment may be attractive to investors due to increasing demand, changing coverage and reimbursement policies, and historically fragmented care delivery. Recent estimates suggest that PE firms now own approximately 1 of 14 substance use treatment facilities.^1^ PE firms typically finance acquisitions with debt placed on the acquired entity and seek high returns over short investment horizons of 3 to 7 years,^3,4^ raising questions about financial incentives that may influence care access, delivery, and outcomes. These new ownership trends are particularly important to understand given the unique intersections between the ongoing opioid epidemic and longstanding treatment access gaps.^5,6^

Research from other areas of health care has prompted concerns about the broader effects of PE ownership, which may be relevant to behavioral health.^3,7,8^ In hospital settings, PE acquisitions have been associated with asset devaluation,^9^ shifts in service mix,^10^ price increases,^11^ and reductions in quality of care.^12^ PE acquisitions of physician practices, which operate under different financial models, have been linked to increased prices and potential shifts in clinical staffing.^13,14,15,16^ While some PE strategies may enhance efficiency, others may change the delivery of care or constrain access, which may be particularly concerning in behavioral health where treatment supply is limited, quality varies widely, and patients are highly vulnerable.^17^

How PE ownership might alter the substance use treatment sector is unknown, particularly in comparison with legacy for-profit facilities. The landscape of SUD treatment in the US is characterized by limited access, long wait times, and substantial heterogeneity in care models.^18,19^ Most individuals in the US face a fragmented market composed of a mix of nonprofit and for-profit facilities.^20^ For-profit facilities comprise a growing proportion of these facilities, increasing from 32% of SUD treatment facilities in 2011 to nearly 50% by 2020.^21^ Whether PE ownership is distinct from legacy for-profit facilities is an important empirical and policy question: Is this new wave of investment an essential feature of dominant for-profit trends or does it introduce new business practices with different implications for patient access and affordability?

To address this gap, we used a national secret shopper approach to compare PE-owned and non-PE, for-profit residential SUD treatment programs on measures of pricing and other facility characteristics that might influence patient access and experience. This approach allows us to capture information directly from facilities as it is presented to potential patients, overcoming limitations of administrative or secondary data sources used in prior studies assessing PE ownership.^1,22,23^ Findings from this analysis may help enhance understanding about trends and impacts of PE ownership in SUD care, as well as inform ongoing state-level policies focused on regulating and monitoring health care ownership shifts.

## Methods

### Overview

This cross-sectional study used a secret shopper framework to assess key measures of price and facility characteristics of residential PE-owned facilities offering SUD treatment in the US compared with geographically matched, non-PE, for-profit controls. This study was approved by the Oregon Health & Science University Institutional Review Board. Informed consent was not required because the study used minimally intrusive simulated patient calls to gather policy-relevant data, an approach considered ethically justified when the risks to participants are minimal and potential societal value is high.^24^ We followed the Strengthening the Reporting of Observational Studies in Epidemiology (STROBE) reporting guideline for cross-sectional studies.

### Facility Identification

PE-acquired residential SUD treatment facilities were identified using Pitchbook and Levin Associates Healthcare M&A data, supplemented with manual searches of websites, press releases, and industry reports, as described in a previous study.^1^ The sample included all facilities acquired between January 1, 2012, and July 31, 2023. Of 833 SUD treatment facilities acquired by PE firms during the study period, 755 (90.6%) were successfully matched to the 2023 Substance Abuse and Mental Health Services Administration National Substance Use and Mental Health Services Survey (N-SUMHSS) using probabilistic matching algorithms, exact matching on street address, and probabilistic (fuzzy) matching on names with subsequent manual verification.

We used the N-SUMHSS as our sampling frame because it is the most comprehensive, nationally administered dataset of SUD and mental health facilities.^25^ The N-SUMHSS includes more than 20 000 eligible SUD and mental health facilities (response rate, 84.9%)^25^ and provides facility-level information (address, phone number), as well as self-reported characteristics such as service offerings, ownership type (for profit, nonprofit, government), and insurance acceptance. Based on facility-reported characteristics in N-SUMHSS, we restricted the sample to residential treatment programs, yielding 151 PE-acquired residential facilities for inclusion. We excluded inpatient psychiatric hospitals and facilities offering only outpatient services.

To identify a comparison group, we restricted N-SUMHSS facilities to non-PE, for-profit residential SUD treatment facilities, further excluding nonprofit and government facilities to ensure comparability.^1^ Each PE facility was matched with up to 2 control facilities based on nearest geographic distance using the FindTreatment.gov locator.^26^ Matching was conducted without replacement, such that each non-PE facility could serve as a control for only 1 PE facility. This approach was selected to avoid overrepresentation of a small number of control facilities and to reduce the potential for artificially precise estimates arising from reuse of controls. In sensitivity analyses, we re-estimated models using matching with replacement to assess the robustness of findings to alternative matching strategies.

### Secret Shopper Procedure

From June 2024 to April 2025, 4 trained secret shoppers (K.H., K.M., D.Z., and S.J.) called facilities using a standardized script adapted from a prior study (eMethods in Supplement 1).^27^ Calls were conducted during standard business hours (9 am-5 pm, Monday-Friday) in the local time zones of each facility. If a call was not completed, a secret shopper attempted follow-up calls on a different day over the next week. Each facility was called up to 3 times. Following data collection, we further excluded facilities that exclusively served populations outside the scope of our caller identity (eg, women-only programs, sexual orientation–specific groups, veterans).

### Study Outcomes

Our primary outcome was price, calculated as a daily rate. We collected data on several secondary outcomes, including accepted payment modalities, as well as facility characteristics, such as wait times, Medicaid acceptance, availability of cost-reduction options, whether payment plans were offered, and bed availability. Additional characteristics included patient eligibility criteria, screening processes, service availability, rooming arrangements and amenities, and facility outreach. We modeled the number of facilities with 0-day wait times to treatment, stratifying these facilities via zip code by rural/urban status using US Department of Agriculture 2023 rural-urban continuum codes.

### Statistical Analysis

Because facilities did not consistently provide responses to all questions, we recorded the total number of responses for each item; rates of missingness are available in eTable 1 in Supplement 1. For numerical variables, we reported means, medians, and IQRs, using 2-sided *t* tests used to test for group differences. For categorical variables, we reported counts and percentages, using χ^2^ or Fisher exact tests as appropriate. We corrected for multiple comparisons using the Benjamini-Hochberg procedure.^28^

To evaluate pricing differences between PE and control facilities, we first used univariable linear regression to model the association of ownership status (PE vs non-PE for-profit) with mean daily price. Because local market conditions could influence facility pricing, we next estimated multivariable linear regression models with fixed effects at the matched cluster level, where each cluster consisted of 1 PE facility and its matched non-PE control or controls, to adjust for local price variation. Our main specification used fixed effects at the matched cluster level, which restricted comparisons to PE facilities and their geographically matched non-PE controls. Finally, we refined these models by incorporating facility-level treatment and amenity offerings (availability of outpatient treatment, luxury accommodations, and medication management) using mixed-effects modeling. A full list of models can be found in eTable 4 in Supplement 1.

All analyses were conducted using Stata version 18.0 (StataCorp LP). All tests were 2-tailed with statistical significance set at *P* < .05.

## Results

In total, 458 calls were conducted to both PE and control facilities, with 450 (98.2%) completed and 394 (86.0%) in the sample (eTables 1 and 2 in Supplement 1). After applying our prespecified facility exclusion criteria and removing duplicates and PE-owned facilities for which we were unable to find suitable controls, our final analytic sample included 127 PE-acquired facilities and 214 non-PE for-profit facilities (mean [SD], 1.69 [0.48] controls per PE facility). These facilities were located in 34 states (Figure).

**Figure.**
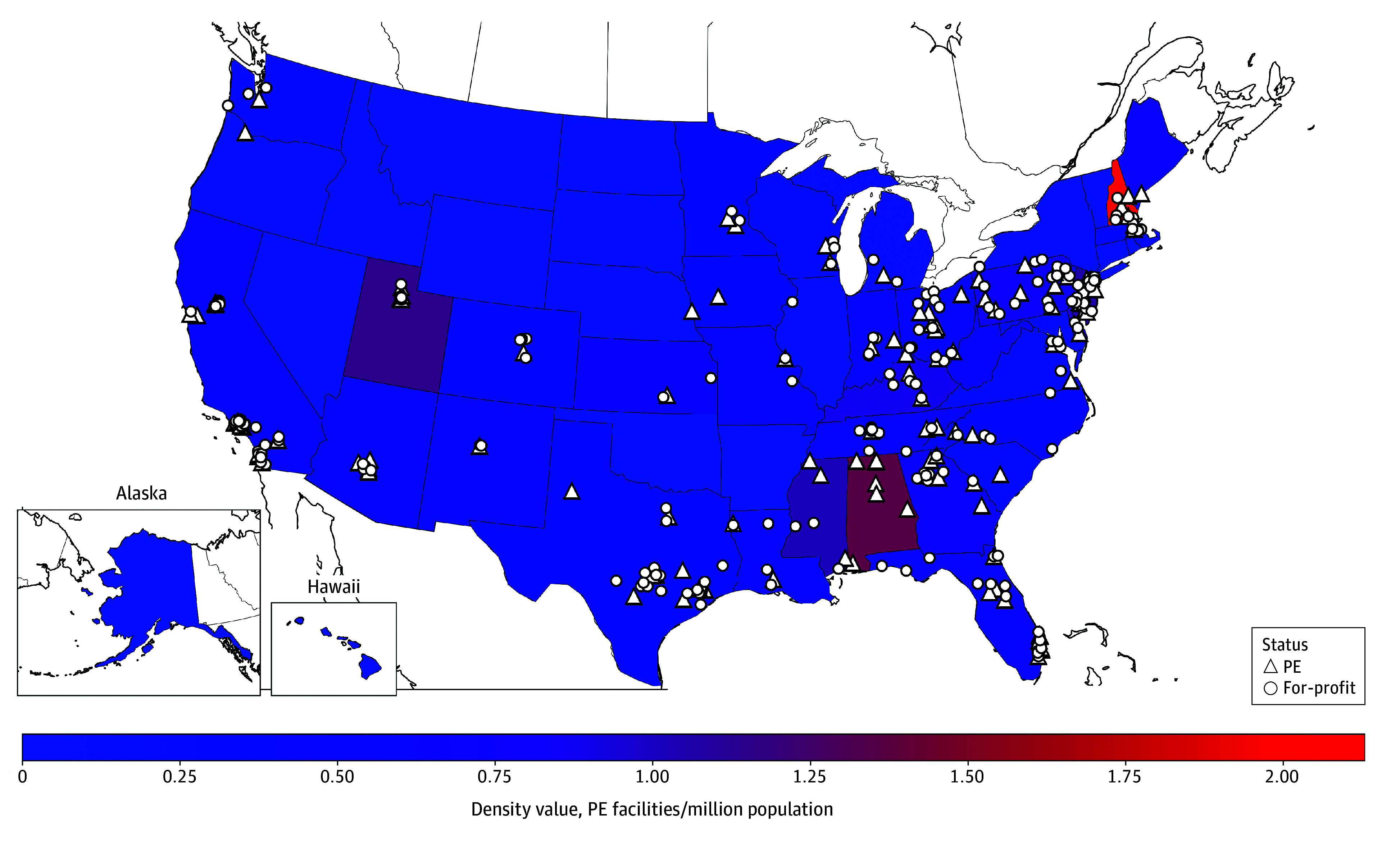
Private Equity (PE)–Owned Substance Use Disorder (SUD) Residential Treatment Facility Locations and Density per Million State Population, 2024 Density values are calculated as the number of PE-owned SUD treatment facilities per million state population.

### Payment Characteristics

Mean (SD) daily rates were 15.6% higher at PE facilities compared with non-PE facilities ($910.73 [$463.16] vs $779.87 [$501.92]; multiple comparisons–adjusted *P* = .04) (Table 1). PE facilities were less likely than non-PE for-profit facilities to accept cash (34.2% vs 54.2%; adjusted *P* = .01) and cashiers’ checks (64.9% vs 79.5%; adjusted *P* = .01) as payment methods. Unadjusted analyses showed that PE facilities charged, on average, $130.85 more per day compared with non-PE for-profit facilities (95% CI, $18.02-$243.68; adjusted *P* = .03) (Table 2). Adjusted models using cluster-matched fixed effects demonstrated that PE facilities charged $127.73 more per day (95% CI, $29.57-$225.87; adjusted *P* = .03). This finding was robust to adjustment for treatment offerings and amenities and across model specifications (eTable 4 in Supplement 1). In sensitivity analyses using our original matching with replacement, estimates were directionally similar to the primary analysis but less precise, with wider CIs (eTable 5 in Supplement 1).

**Table 1.** Comparison of Payment Characteristics Between PE-Acquired and Non-PE For-Profit SUD Residential Treatment Facilities, 2024^a^

Payment characteristics	No. of PE facilities (% of responding)	No. of non-PE for-profit facilities (% of responding)	*P* value for difference	*P* value (adjusted for multiple comparisons)
Daily rate, $				
Mean (SD)	910.73 (463.16)	779.87 (501.92)	.02^b^	.04^b^
Median (IQR)	854.29 (589.29-1071.43)	750.56 (480.64-952.38)		
Payment modality accepted				
Cash	39 (34.2)	103 (54.2)	.03^b^	.03^b^
Credit/debit	113 (99.1)	183 (96.3)	.14	.17
Loans	0 (0.0)	3 (1.6)	.30	.30
Cashiers’ check	74 (64.9)	151 (79.5)	.03^b^	.03^b^

Abbreviations: PE, private equity; SUD, substance use disorder.

^a^ Differences in categorical variables were assessed using χ^2^ test; differences in continuous variables (mean daily rate) were assessed using *t* test. Data on response rates by ownership status for each variable are available in eTable 3 in Supplement 1; missingness varied by <5% between PE and for-profit firms for nearly all variables. Adjusted *P* values were adjusted for multiple comparisons at the table level using the Benjamini-Hochberg procedure.

^b^ Denotes α < .05 on χ^2^ test (categorical variables) and *t* test (continuous variables).

**Table 2.** Association Between PE Ownership and Mean Daily Rate Across Model Specifications^a^

Model	Specification	Market fixed effects	Services adjustment	No.	*F*-score/χ^2^	β (95% CI)^b^	*P* value	*P* value (adjusted for multiple comparisons)
1	Unadjusted difference, mean daily rate, PE-owned less non-PE for-profit facilities	No	No	313	5.21	130.85 (18.02-243.68)	.02^c^	.03^c^
2	Linear regression, PE ownership on mean daily rate, with market fixed effects	Yes	No	313	6.63	127.73 (29.57-225.87)	.01^c^	.03^c^
3	Model 2, further adjusted for differences in service offerings	Yes	Yes	302	1.71	120.98 (14.82-227.14)	.03^c^	.03^c^

Abbreviation: PE, private equity.

^a^ The outcome variable in all models is the facility-reported mean daily rate in dollars. PE ownership was defined using a binary indicator (1 = PE-owned, 0 = non-PE for-profit). Model 1 reports the unadjusted difference in mean daily rate between PE-owned and non-PE for-profit facilities (model results noted in Table 1). Model 2 estimates the association between PE ownership and daily rate using a linear regression model with matched-cluster fixed effects, comparing facilities within the same geographic market. Model 3 extends this specification by adjusting for observable differences in service and amenities, including availability of outpatient treatment, luxury accommodations, and medication management. All models exclude facilities with missing cost data. Robust SEs were used to calculate 95% CIs. Adjusted *P* values were adjusted for multiple comparisons at the table level using the Benjamini-Hochberg procedure.

^b^ The β coefficient is the dollar value; regressed on price per day.

^c^ Denotes α < .05 on regression analysis.

### Facility Characteristics

Compared with 74.6% (153 of 205) of non-PE for-profit facilities, 64.3% (74 of 115) of PE facilities reported an available bed (Table 3). Available beds were similar between PE and non-PE facilities in both rural (60.0% [9 of 15] vs 69.6% [16 of 23]) and urban (65.0% [65 of 100] vs 75.3% [137 of 182]) areas (eFigure 1 in Supplement 1). Approximately one-third of facilities reported accepting Medicaid, with no statistically significant difference between PE (34.1% [43 of 126]) and non-PE for-profit facilities (38.3% [82 of 214]) (Table 3). Nearly all facilities required some amount of upfront payment, and fewer than one-quarter of facilities across both groups offered cost-reduction options. PE facilities were less likely to offer on-site detox services (74.8% [95 of127] vs 88.8% [190 of 214]; adjusted *P* = .02) and to offer private rooms (12.1% [15 of 124] vs 25.7% [54 of 210]; adjusted *P* = .02) (Table 3), while the proportion offering luxury amenities and proportion of facilities offering travel assistance did not differ significantly between groups.

**Table 3.** Comparison of Facility Characteristics Between PE-Acquired and Non-PE For-Profit SUD Residential Treatment Facilities, 2024^a^

Characteristics	No. of PE facilities (% of responding)	No. of non-PE for-profit facilities (% of responding)	*P* value for difference	*P* value (adjusted for multiple comparisons)
Bed available	74 (64.3)	153 (74.6)	.05	.13
Accepts Medicaid	43 (34.1)	82 (38.3)	.44	.59
Upfront payment required	109 (92.4)	175 (89.7)	.44	.59
Cost-reduction options available	28 (23.9)	48 (23.9)	.99	.99
Payment plan available	55 (48.3)	70 (36.5)	.04^b^	.13
Wait for intake, median (IQR), d	0 (0-2)	0 (0-1)	.99	.99
Dual diagnosis emphasis	74 (58.3)	124 (57.9)	.95	.99
Drug testing required prior to admission	56 (45.9)	115 (56.1)	.07	.15
Criminal history disqualifying	27 (21.4)	27 (13.4)	.06	.13
Detox available	95 (74.8)	190 (88.8)	.001^b^	.02^b^
Medication management for psychiatric comorbidities	70 (56.5)	96 (45.3)	.05^b^	.13
Psychiatrist on staff	115 (93.5)	177 (86.3)	.05^b^	.13
Counseling and therapy available	118 (95.2)	208 (98.4)	.12	.20
Private rooms	15 (12.1)	54 (25.7)	.003^b^	.02^b^
Luxury amenities (eg, pool, exercise)	72 (61.0)	108 (51.4)	.09	.17
Travel assistance available	93 (75.6)	154 (78.2)	.60	.73

Abbreviations: PE, private equity; SUD, substance use disorder.

^a^ Differences in categorical variables assessed with χ^2^ test; differences in continuous variables assessed with *t* test. Data on response rates by ownership status for each variable are available in eTable 3 in Supplement 1; missingness varied by <5% between PE and for-profit firms for nearly all variables. Adjusted *P* values were adjusted for multiple comparisons using the Benjamini-Hochberg procedure at the table level.

^b^ Denotes α <.05 using χ^2^ test (categorial variables) and *t* test (continuous and pseudocontinuous variables).

### Facility Outreach

Following the initial secret shopper inquiry, 44.1% of PE facilities and 37.9% of non-PE facilities attempted to follow-up with the caller (eTable 6 in Supplement 1). PE facilities made significantly more follow-up contact attempts than non-PE facilities, with a mean (SD) of 0.68 (1.39) vs 0.18 (0.47) attempts (adjusted *P* < .001).

## Discussion

Against the backdrop of rapidly expanding PE investment in behavioral health, this national secret shopper study provides a unique lens into how ownership influences what patients encounter when seeking residential SUD treatment. Our findings suggest some notable differences between PE-acquired residential SUD treatment facilities and geographically matched, non-PE for-profit facilities across several dimensions. Most notably, in adjusted analyses accounting for geographic clustering and observable service offerings, PE facilities charged $127 more per day than non-PE for-profit controls. In secondary descriptive analyses, PE facilities were less likely to offer detox services, less likely to offer private rooms, and more likely to follow-up with potential patients after an inquiry. These findings highlight the need for future work to assess whether these differences reflect variation in costs, quality, or market dynamics, and to evaluate their implications for access and patient outcomes.

Our finding of higher daily rates aligns with prior studies documenting price increases after PE acquisitions of physician practices and hospitals.^11,14^ At the same time, PE facilities were less likely to report certain amenities, such as private rooms, suggesting differences in facility offerings rather than uniformly higher observable inputs. Our data were not able to fully capture variation in service mix, staffing models, quality, or underlying cost structures that may explain some of these price differences. At the same time, PE-owned facilities appear to recruit post-outreach more frequently than non-PE for-profit peer facilities, reaching out more than 3 times as much after calls, which suggests potential operational differences in patient engagement or intake practices. These findings are consistent with a recent descriptive study on PE-owned psychiatric hospitals, which found higher occupancy and lower staffing levels.^23^ The implications of these findings for patient care remain uncertain.

Differences in other operational and facility characteristics, such as follow-up practices, room configurations, and service offerings, are descriptive and should be interpreted cautiously. More-frequent follow-up for inquiring patients may reduce barriers to information for patients navigating a fragmented referral system, while higher room occupancy may reflect efforts to maximize fixed capacity. At the same time, these patterns may also reflect differences in business strategy, staffing models, or cost structures that are not directly observable in a cross-sectional design. Prior research in other health care sectors, such as PE-owned hospitals and nursing homes, shows that such operational adjustments can alter how resources are allocated across patients,^11,29^ raising questions about whether similar trade-offs may be occurring in residential SUD care.

These findings also carry policy relevance. The prevalence of SUD increased during the COVID-19 pandemic, yet the receipt of SUD treatment has continued to be low^30^ despite resumption of health care services and increased telehealth use. States including New Mexico,^31^ Massachusetts,^32^ and Oregon^33^ have recently taken steps aimed at increasing oversight of PE ownership in health care, although regulatory approaches vary. In Oregon, for example, lawmakers recently created new restrictions on corporate ownership of medical practices, but excluded behavioral health facilities due to concerns about access constraints. Our results suggest that PE-owned residential SUD facilities differ from other for-profit facilities primarily in reported prices, charging $127 more per day on average, with additional differences in select facility and operational characteristics. While this study cannot determine whether higher prices reflect differences in quality, costs, or market power, the findings underscore the importance of including behavioral health settings in ongoing monitoring and data collection efforts. State and federal policymakers assessing the implications of ownership structure for affordability and access should include behavioral health facilities in their evaluations.

### Limitations

This study has several limitations. First, as with other research in this area, we relied on multiple sources to confirm PE ownership status and misclassification is possible despite manual reviews. Second, we were unable to capture certain important aspects of patient care, such as pharmacotherapeutic offerings or first-person patient experience, which represent targets for future research. Our analysis was also restricted to for-profit residential SUD treatment facilities and did not include nonprofit, outpatient only, or inpatient facilities, which limits generalizability across the broader treatment system. Third, because our design relied on a secret shopper approach, findings reflect information provided to prospective patients during intake calls, which may not align with services delivered during treatment. In addition, the cross-sectional nature of our study precludes causal inference, and we cannot determine whether the observed differences were directly caused by PE ownership or instead reflect selection or other unmeasured factors. Fourth, some data elements had relatively high rates of missingness. While we report the extent of missing data transparently, missingness could bias our results. Fifth, PE facilities may exert pricing pressure or influence on nearby non-PE facilities, potentially attenuating observed differences across a variety of characteristics we report. While our analyses include clustered market-level fixed effects to account for shared local factors, this approach will not capture effects of within-market spillovers. As a result, our estimates may understate true effects of PE. Finally, our study did not include patient outcomes, which remains an essential direction for future work.

## Conclusions

This study provides new descriptive evidence on how PE-acquired residential SUD facilities differ from their non-PE for-profit counterparts in the US. Using a novel comparison group and a secret shopper design, we found that PE-acquired facilities charged, on average, $127 more per day compared with non-PE for-profit facilities, with differences in selected facility characteristics. As PE continues to expand its footprint in behavioral health, understanding these dynamics and their impacts will be important for addressing an ongoing national substance use crisis. Further work using longitudinal designs and patient outcomes data is needed to assess the mechanisms underlying these differences and their implications for affordability and quality of care.

## Notes

Supplement 1. **eMethods. **Additional detailed methods including secret shopper call script **eTable 1. **Call completion by ownership status **eTable 2. **Count of controls for each PE facility **eTable 3. **Response rates by question **eTable 4. **Sensitivity analyses: Association between PE ownership and daily cost under alternative model specifications **eTable 5. **Sensitivity analyses: Association between private equity ownership and daily cost under alternative matching strategy (Matching with replacement) **eTable 6. **Post-call contact attempts by PE facilities vs non-PE for-profit controls **eFigure 1. **Wait times by facility type, by rural and urban status of county

Supplement 2.
Data Sharing Statement
