# Supplementary material for: Private Equity–Acquired Residential Treatment Facilities vs Other For-Profit Facilities
Source: JAMA Health Forum. 2026 Apr 3;7(4):e260414. doi: 10.1001/jamahealthforum.2026.0414 (PMC13049487; doi:10.1001/jamahealthforum.2026.0414)
Supplement: Supplement 1. — eMethods. Additional detailed methods including secret shopper call script eTable 1. Call completion by ownership status eTable 2. Count of controls for each PE facility eTable 3. Response rates by question eTable 4. Sensitivity analyses: Association between PE ownership and daily cost under alternative model specifications eTable 5. Sensitivity analyses: Association between private equity ownership and daily cost under alternative matching strategy (Matching with replacement) eTable 6. Post-call contact attempts by PE facilities vs non-PE for-profit controls eFigure 1. Wait times by facility type, by rural and urban status of county [file jamahealthforum-e260414-s001.pdf]

Supplemental Online Content

Havlik JL, Busch S, Hidalgo K, Mercado K, Zhu D, Jensen S, Zhu JM. Private equity–acquired residential treatment facilities vs other for-profit facilities. *JAMA Health Forum*. 2026;7(4):e260414. doi:10.1001/jamahealthforum.2026.0414

eMethods. Additional detailed methods including secret shopper call script

eTable 1. Call completion by ownership status

eTable 2. Count of controls for each PE facility

eTable 3. Response rates by question

eTable 4. Sensitivity analyses: Association between PE ownership and daily cost under alternative model specifications

eTable 5. Sensitivity analyses: Association between private equity ownership and daily cost under alternative matching strategy (Matching with replacement)

eTable 6. Post-call contact attempts by PE facilities vs non-PE for-profit controls

eFigure 1. Wait times by facility type, by rural and urban status of county

This supplemental material has been provided by the authors to give readers additional information about their work.

© 2026 Havlik JL et al. *JAMA Health Forum*.

eMethods

Control facility identification. We determined control for-profit non-PE facilities using the SAMHSA website FindTreatment.gov (https://findtreatment.gov/). Filters were set to include only for-profit facilities that offered substance use disorder (SUD) treatment, were not classified as PE-owned in our N-SUMHSS dataset, and provided residential treatment services.

Each PE-owned facility was paired with potential comparators located within a 10-mile radius. If fewer than the required number of control facilities were available, the search radius was expanded stepwise to 15 miles, 25 miles, and, if necessary, to the state level. When no eligible comparators were identified at the state level, we assigned an available control from a neighboring state. Otherwise, no control was assigned and the PE-owned facility was excluded from analysis.

Secret shopper call script

(Adapted from: Beetham et al (2021) Admission Practices and Cost at Residential Addiction Treatment Programs in the USA: National Audit Survey)

Introduction/backstory:

Hello, my name is [Name], and I'm calling about my brother. He's been using oxycodone and dilaudid for a couple of years and had an overdose in February. He's still using but he's willing to get help and I'm trying to find the best treatment option for him. Can you help me with some questions about your center?

Background: Brother has never been to treatment center before, he has only gotten suboxone for detox in ED. Brother is a Hispanic male, 22 years old, using for two years. He has found it difficult to stop before on his own because he gets triggered by things that remind him of using pills or stresses in his life or at work. His use started when some of his friends in college introduced him to pain pills. He subsequently dropped out of school and is now living with his mom and actively using. He has never injected anything, he hates needles. He doesn't have a history of HIV or justice involvement.

Questions:

Payment and Insurance:

- Do you accept patients without insurance?
  - [If yes] Are there additional costs not covered by insurance? (Outcome: Yes/No/Unsure/Treatment is free)
- Do you accept Medicaid for treatment? I think he might have been getting set up with that but I am not yet sure if it went through (Outcome: Yes/No/Unsure)

Costs:

- What is the cost for the treatment?
  - How many days does this cover? (Outcome: Cost/Number of days)
- How do people usually pay?

© 2026 Havlik JL et al. *JAMA Health Forum*.

  - To help get money together, I need to know if you accept cash, cashier's check, money order, or if there are other ways to pay. (Outcome: Cash/Cashier's Check/Money Order/Other)
- Is there anything we need to know about upfront costs? Like, do we need to have all the money upfront, or can we pay a portion of it? (Outcome: 100%/Other/Unsure)
- Are there any options to help with the cost- some friends told me that sometimes there are payment plans, price reduction, and maybe some public grants? (Outcome: Negotiable/No Payment Plan/Price Reduction/Public Grants/Scholarships/Don't know or talk to billing)
- If he gets Medicaid, how does the cost change?
- What if I helped to cover some of the costs? How would that work?

Facility characteristics:

- How soon could he get a bed? (Outcome: Days)
- Is there a way to know if he can be admitted now? How does the process work? (Outcome: Yes, after initial screening/No, waitlist/Depends on further screening)
- Do you offer suboxone? [y/n]
  - [If yes], is it used for maintenance long-term? (Outcome: Yes/No/Unsure)
- Are there any issues that might be disqualifying for admission?
  - Is there anything else that could prevent him from getting admitted, like his medical or legal history or something? (Outcome: Record psychiatric history/Medical history/Financial screening/Legal/Can't disclose)
- I think he's really depressed and has bad anxiety. Could the facility treat his depression and anxiety? (Outcome: Yes/No/Unsure/Depends)
- Is there any psychotherapy available as part of the treatment? (Outcome: Yes/No/Unsure/Depends)
- He's never been to a treatment facility before and he's worried about safety. What does the facility do to make sure this is a safe environment?
- We're also looking at a few other places – do you offer any additional amenities that sets you apart?
- My brother might have some trouble getting to your location. Does your facility help with arranging transportation or provide travel support? (Outcome: Facility pays for car ride/Facility books or pays for flight/Facility helps to arrange or support travel/Other general transportation resources available)
- Do you feature other amenities, like single rooms? (Outcome: Yes/No/Describe. Probe: rooming, pool, facilities, food, recreation)
- Is there anything else I didn't ask that I should tell him when I review these options with him?

© 2026 Havlik JL et al. *JAMA Health Forum*.

eTable 1. Call completion by ownership status. (call observation – not at the facility level)

		Call observation					Facility level
	Calls attempted	Calls completed	Exclusions	In-sample	Duplicate controls (removed)	No unique controls (removed)	# of unique facilities
PE	151	150	7	143	N/A	16	127
Controls	307	300	49	251	37	N/A	214

Control facilities were facilities offering residential substance use disorder treatment that were not PE-owned as per our dataset. We initially searched findtreatment.gov for facilities were within 10 miles of each PE-owned comparator facility. Where an inadequate number of facilities were found, we expanded our search radius from 10 to 15 mile, then 25 miles, etc., expanding our search to the state level as needed. If no suitable comparator facilities were identified even at the state level, we expanded our search to neighboring states.

© 2026 Havlik JL et al. *JAMA Health Forum*.

eTable 2. Count of controls for each PE facility

	# of controls		
	1	2	3
Count of PE facilities	40	86	1

Two controls attempted for each PE facility. Duplicate controls were permitted whereby one control could be used for multiple PE facilities. PE facilities with only 1 control were due to geographic or population restrictions and other exclusions after completed calls. One facility (with third control called mistakenly) with three controls included in sample.

© 2026 Havlik JL et al. *JAMA Health Forum*.

eTable 3. Response rates by question

	**PE (n = 127) (%)**	**Non-PE For-Profit (n = 214) (%)**
Bed available*	115 (90.6)	205 (95.8)
Accepts Medicaid	126 (99.2)	214 (100.0)
Mean wait time for intake*	115 (90.6)	205 (95.8)
Dual diagnosis emphasis	127 (100.0)	214 (100.0)
Drug testing required prior to admission	122 (96.1)	205 (95.8)
Criminal history disqualifies admission*	126 (99.2)	201 (93.9)
Detox available	127 (100.0)	214 (100.0)
Medication management offered for psychiatric comorbidities	124 (97.6)	212 (99.1)
Psychiatrist on staff	123 (96.9)	205 (95.8)
Mental health counseling and therapy available	124 (97.6)	212 (99.1)
Private rooms	124 (97.6)	210 (98.1)
Luxury amenities (e.g., pool, exercise)*	118 (92.9)	210 (98.1)
Travel assistance available	123 (96.9)	197 (92.1)
Mean daily rate	114 (89.8)	199 (93.0)
Payment modality accepted		
Cash	114 (89.8)	190 (88.8)
Credit/Debit	114 (89.8)	190 (88.8)
Loans	114 (89.8)	190 (88.8)
Cashiers' check	114 (89.8)	190 (88.8)
Upfront payment required	118 (92.9)	195 (91.1)
Cost reduction options available	117 (92.1)	201 (93.9)
Payment plan available	114 (89.8)	192 (89.7)

Variables with more than 5% missingness differences across ownership groups denoted with *

© 2026 Havlik JL et al. *JAMA Health Forum*.

eTable 4. Sensitivity analyses: Association between PE ownership and daily cost under alternative model specifications

Model	1	2 (main)	3	4	5	6
**Specification**	Unadjusted difference, mean daily rate, PE-owned less non-PE for-profit facilities	**Linear regression, PE ownership on mean daily rate, with market fixed effects**	Model 2, further adjusted for differences in service offerings	Model 1, SE adjusted for clustering at geographic level	Model 1, adjusted for clustering at geographic level and differences in service offerings	Model 2, random instead of fixed effects
**Market FEs**		Y	Y			
**Services adjustment**			Y		Y	
**Adjusted for market clustering**				Y	Y	
**N**	313	313	302	313	302	302
**F-score/chi squared**	5.21	6.63	1.71	7.13	1.50	9.15 (chi squared)
**Est (beta)**	130.85	127.73	120.98	130.85	135.79	130.12
**95% CI**	(18.02, 243.68)	(29.57, 225.87)	(14.82, 227.14)	(33.88, 227.82)	(18.07, 253.51)	(45.82, 214.42)
**P-value**	**0.023***	**0.011***	**0.026***	**0.009***	**0.024***	**0.002***
**P-value (adjusted for multiple comparisons)**	**0.026***	**0.022***	**0.026***	**0.022***	**0.026***	**0.012***

Outcome variable in all models is the mean reported daily cost of care in dollars ($). PE ownership was identified using a binary indicator equal to 1 if the facility is owned by a private equity firm, and 0 otherwise. Model 1 reports unadjusted difference in mean daily cost between PE-owned and non-PE for-profit facilities. Model 2 estimates the association between PE ownership and cost per day using a linear regression model with market fixed effects, comparing facilities within the same geographic market (using a matched cohort identifier). Model 3 extends the fixed effects model by including adjustments for observable differences in service offerings including facility reported outpatient treatment, luxury amenities, and medication management. Model 4 adjusts Model 1 standard errors (SEs) for geographic clustering. Model 5 further adjusts Model 4 for differences in service offerings highlighted above in this caption. Model 6 recasts the fixed effects model of Model 2 as a random effects model. All models exclude facilities with missing rate data. Confidence intervals are based on robust standard errors. * Denotes $\alpha < 0.05$ on regression analysis.

© 2026 Havlik JL et al. *JAMA Health Forum*.

eTable 5. Sensitivity analyses: Association between private equity ownership and daily cost under alternative matching strategy (Matching with replacement)

Model	1	2 (main)	3	4	5	6
**Specification**	Unadjusted difference, mean daily rate, PE-owned less non-PE for-profit facilities	**Linear regression, PE ownership on mean daily rate, with market fixed effects**	Model 2, further adjusted for differences in service offerings	Model 1, SE adjusted for clustering at geographic level	Model 1, adjusted for clustering at geographic level and differences in service offerings	Model 2, random instead of fixed effects
**Market FEs**		Y	Y			
**Services adjustment**			Y		Y	
**Adjusted for market clustering**				Y	Y	
**N**	334	334	321	334	321	334
**F-score/chi squared**	3.04	5.27	2.18	4.29	1.03	5.14 (chi squared)
**Est (beta)**	96.54	101.03	91.9	96.54	98.93	99.10
**95% CI**	(-12.46, 205.5)	(14.29, 187.76)	(-1.72, 185.52)	(4.28, 188.81)	(-15.67, 213.52)	(13.42, 184.87)
**P-value**	0.082	**0.023***	0.054	**0.040***	0.090	**0.023***
**P-value (adjusted for multiple comparisons)**	0.090	0.069	0.081	0.080	0.090	0.069

Outcome variable in all models is the mean reported daily cost of care in dollars ($). PE ownership was identified using a binary indicator equal to 1 if the facility is owned by a private equity firm, and 0 otherwise. Model 1 reports unadjusted difference in mean daily cost between PE-owned and non-PE for-profit facilities. Model 2 estimates the association between PE ownership and cost per day using a linear regression model with market fixed effects, comparing facilities within the same geographic market (using a matched cohort identifier). Model 3 extends the fixed effects model by including adjustments for observable differences in service offerings including facility reported outpatient treatment, luxury amenities, and medication management. Model 4 adjusts Model 1 standard errors (SEs) for geographic clustering. Model 5 further adjusts Model 4 for differences in service offerings highlighted above in this caption. Model 6 recasts the fixed effects model of Model 2 as a random effects model. All models exclude facilities with missing rate data. Confidence intervals are based on robust standard errors. * Denotes $\alpha < 0.05$ on regression analysis.

© 2026 Havlik JL et al. *JAMA Health Forum*.

eTable 6. Post-call contact attempts by PE facilities vs non-PE for-profit controls

Variable	PE	For-Profit	P-value	P-value (adjusted for multiple comparisons)
Any attempt to contact post-call	56 (44.1)<br>*n=127*	81 (37.9)<br>*n= 214*	0.152	0.228
Attempt to email/text post-call	25 (19.7)<br>*n=127*	40 (18.7)<br>*n= 214*	0.488	0.488
Mean number of contact attempts, (Median, IQR)	0.68 (0 [0-1])<br>*n=127*	0.18 (0 [0-0])<br>*n= 214*	**<0.001***	**<0.001***

IQR: Interquartile range. Adjusted p-values were adjusted for multiple comparisons using the Benjamini-Hochberg procedure. * Denotes α < 0.05 on chi-squared testing (categorial variables) and t-testing (continuous and pseudo-continuous variables).

© 2026 Havlik JL et al. *JAMA Health Forum*.

eFigure 1. Wait times by facility type, by rural and urban status of county

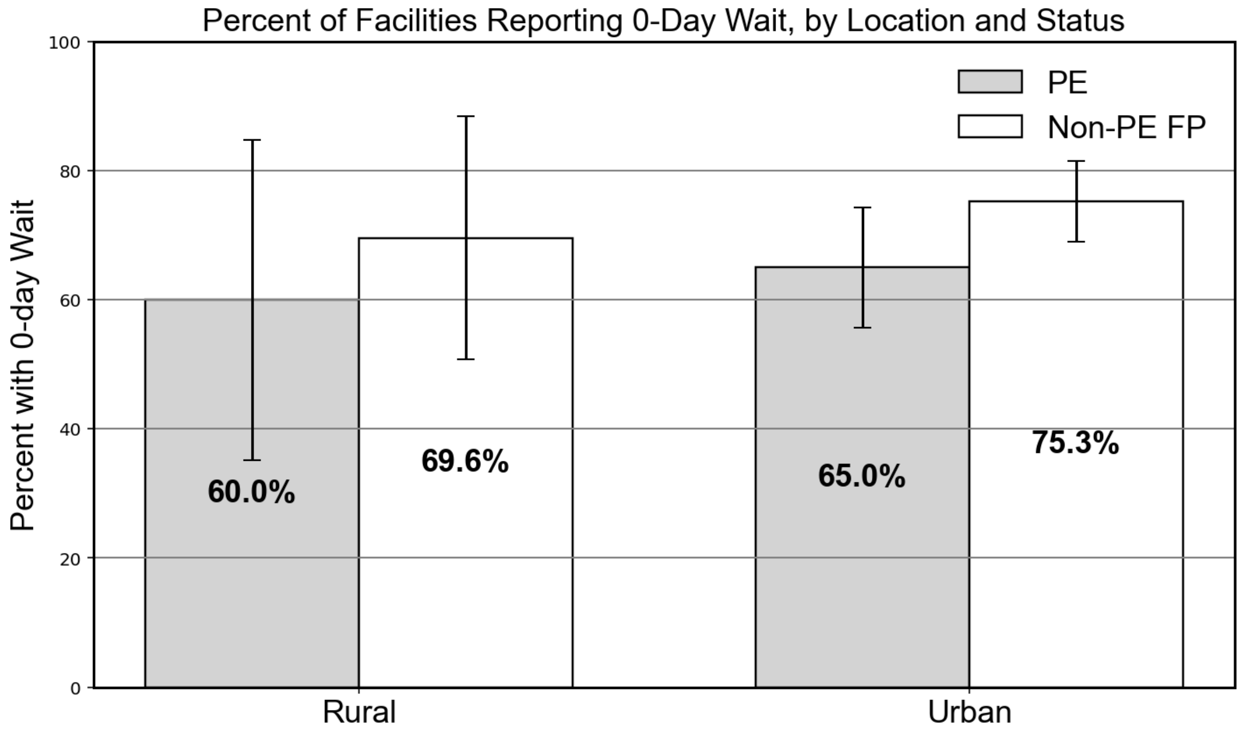

FP: for-profit; PE: private equity. Error bars indicate standard errors. Rural status defined using U.S. Department of Agriculture 2023 rural-urban continuum code (RUCC); a facility located in county with a RUCC greater than 3 was defined as rural. Differences assessed with chi-squared testing (not significant for either comparison).

© 2026 Havlik JL et al. *JAMA Health Forum*.
