# Supplementary material for: Private Equity–Acquired Residential Treatment Facilities vs Other For-Profit Facilities
Source: JAMA Health Forum. 2026 Apr 3;7(4):e260414. doi: 10.1001/jamahealthforum.2026.0414 (PMC13049487; doi:10.1001/jamahealthforum.2026.0414)
Supplement: Supplement 2. — Data Sharing Statement [file jamahealthforum-e260414-s002.pdf]

Data Sharing Statement

Havlik. Private Equity–Acquired Residential Treatment Facilities vs Other For-Profit Facilities. *JAMA Health Forum*. Published April 03, 2026. doi:10.1001/jamahealthforum.2026.0414

Data
Data available: No
